# Regulator of G-Protein Signaling 5 Reduces HeyA8 Ovarian Cancer Cell Proliferation and Extends Survival in a Murine Tumor Model

**DOI:** 10.1155/2012/518437

**Published:** 2012-06-25

**Authors:** Molly K. Altman, Duy T. Nguyen, Santosh B. Patel, Jada M. Fambrough, Aaron M. Beedle, William J. Hardman, Mandi M. Murph

**Affiliations:** ^1^Department of Pharmaceutical and Biomedical Sciences, College of Pharmacy, Pharmacy South Building, The University of Georgia, Athens, GA 30602, USA; ^2^The University of Georgia Medical Partnership, 279 Williams Street, Athens, GA 30602, USA

## Abstract

The regulator of G-protein signaling 5 (RGS5) belongs to a family of GTPase activators that terminate signaling cascades initiated by extracellular mediators and G-protein-coupled receptors. RGS5 has an interesting dual biological role. One functional RGS5 role is as a pericyte biomarker influencing the switch to angiogenesis during malignant progression. Its other functional role is to promote apoptosis in hypoxic environments. We set out to clarify the extent to which RGS5 expression regulates tumor progression—whether it plays a pathogenic or protective role in ovarian tumor biology. We thus constructed an inducible gene expression system to achieve RGS5 expression in HeyA8-MDR ovarian cancer cells. Through this we observed that inducible RGS5 expression significantly reduces *in vitro* BrdU-positive HeyA8-MDR cells, although this did not correlate with a reduction in tumor volume observed using an *in vivo* mouse model of ovarian cancer. Interestingly, mice bearing RGS5-expressing tumors demonstrated an increase in survival compared with controls, which might be attributed to the vast regions of necrosis observed by pathological examination. Additionally, mice bearing RGS5-expressing tumors were less likely to have ulcerated tumors. Taken together, this data supports the idea that temporal expression and stabilization of RGS5 could be a valuable tactic within the context of a multicomponent approach for modulating tumor progression.

## 1. Introduction

The regulator of G-protein signaling 5 (RGS5) belongs to a family of GTPase activators and signal transduction molecules that negatively regulate the function of G-proteins. In other words, RGS proteins terminate cellular signaling cascades initiated by extracellular mediators that bind to and activate G-protein-coupled receptors. More specifically, RGS5 binds to G alpha (i), (q), and (o) subunits within heterotrimeric G-proteins to terminate signaling and is located along the plasma membrane and within the cytosol [1]. RGS5 was isolated in 1997 from mouse pituitary, although its preferential expression is in the heart (particularly aorta), skeletal muscle, lung, small intestine, and thyroid [2, 3]. *Rgs5* is located at 1q23.1, a region on chromosome 1 of interest for lipid metabolism [4], hypertension [5, 6], blood pressure regulation [7], severity of schizophrenia symptoms [8], and association with SNPs that have specific effects dependent upon genetic background [9].

Using a platelet-derived growth factor knockout mouse model and comparing it to the gene expression of wildtype mice, Bondjers et al. were the first to identify RGS5 as a biomarker of pericytes [10], cells that wrap around the walls of capillaries. Pericytes are thus involved in the regulation of blood flow and the transformation of new blood vessels. Berger et al. verified that RGS5 is an angiogenic pericyte marker and a component involved in the switch to angiogenesis during malignant progression [11], with context-specific expression (i.e., during wound healing). Mitchell et al. confirmed that the expression of RGS5 is temporally upregulated during pathological angiogenesis, at approximately 5-6 days after corneal scraping, a period when the nascent vessel sprouts acquire their pericyte covering [12].

Looking more broadly at the gene expression of RGS5 in malignant tumors produces mixed results. Nearly an equivalent number of microarray expression experiments archived in the European Bioinformatics Institute Atlas report that the gene is overexpressed or underexpressed. The interpretation of these mixed results portrays a complex association with intratumor heterogeneity, where gene expression is highly dependent on location [13] and in the specific example of RGS5, also on several other factors, including hypoxia and vascular remodeling.

However, other reports offer more clearly defined explanations. For example, a study by Silini et al. showed that a low level (<1%) of RGS5 fluorescence covered the normal ovary, whereas RGS5 increased to 7.3% coverage in ovarian carcinoma specimens from patient biopsies. Furthermore, the staining pattern of RGS5 coincided with vessel-like structures, which is suggestive of a biomarker for cancer vasculature and consistent with RGS5 expression predominantly resulting from the vascular endothelium of carcinoma [14]. Therefore, RGS5 levels could be expected to vary according to the extent and stage of tumor vascularization, perhaps explaining the gene expression variability for RGS5 among single-biopsy specimens.

Not surprisingly given its association with the vasculature, RGS5 is also significantly affected by hypoxia, which is a response initiated by cells in low-oxygen environments that would otherwise succumb to toxic anoxia and cell death. Cancer cells in solid tumors are notorious for adapting to hypoxic environments by downregulating mitochondrial function [15] and shifting to aerobic glycolysis [16]. Interestingly, Jin et al. showed that endothelial cells exposed to a hypoxic environment (<1% oxygen) display an increase in both mRNA and protein expression of RGS5 beginning at 30 min after exposure and tapering off around 24 hours. Furthermore, they identified RGS5 as a hypoxia-inducible gene that stimulates apoptosis and is regulated by the alpha subunit hypoxia-inducible factor-1 (HIF-1*α*) [17]. The HIF-1 heterodimeric transcription factor is an important regulator of angiogenesis because it causes the expression of numerous target genes (VEGF, PDGF, TGF-*α*, PDK1, COX4I2, etc.) which are involved in neovascularization, erythropoiesis, glucose transport, and energy metabolism [18]. Overexpression of VEGF and HIF-1*α* has been associated with a poor prognosis in ovarian cancer and breast cancer patients [19]. 

Further studies using an RGS5-deficient RIP1-Tag5 transgenic mouse model demonstrated an increased rate of tumorigenesis and reduction in the overall survival of mice through the development of a normalized network of blood vessels, decreased hypoxia and reduced vessel permeability [20–22]. Interestingly, Hamzah et al. also reported that RGS5 is “a master gene responsible for the abnormal tumor vascular morphology in mice” [20]. We thus questioned whether the inducible expression of RGS5 in a tumor model of ovarian cancer might counter the effects observed in knockout mice and support a longer period of survival *in vivo. *Since the role of angiogenesis inhibitors is somewhat controversial (see Section 4 for more details), such a study may also clarify the extent to which RGS5 expression regulates tumor progression, perhaps via altered vascularization. Herein, we observed an increase in the survival time in mice bearing tumors with RGS5 expression coupled with increased areas of necrosis and a reduction in tumor ulceration. The control animals with tumors expressing the vector alone displayed more malignant cells within tumors and more had ulcerated tumors. Although all animals eventually succumb to disease, these studies are suggestive that RGS5 expression reduces malignancy in tumors, thus increasing survival time, and this is independent of its role in vascular normalization and remodeling.

## 2. Methods

### 2.1. Materials

HeyA8-MDR taxane-resistant line of cells were previously described [23] and are maintained in RPMI 1640 medium (Mediatech, Inc., Manassas, VA, USA) supplemented with 300 ng/mL paclitaxel (Sigma-Aldrich, St. Louis, MO, USA) with 15% fetal bovine serum (PAA Laboratories, Inc., Etobicoke, ON, Canada). Approved fetal bovine serum (Clontech Inc., Mountain View, CA, USA) was used in the medium for the pTet Dual RGS5-modified HeyA8-MDR cells. Doxycycline Hyclate was used to suppress gene expression in the Tet-Off system (Sigma-Aldrich, St. Louis, MO, USA). The compounds nocodazole, BrdU, etoposide, and aphidicolin Assay Kit were purchased from Millipore (Billerica, MA, USA).

### 2.2. Construction of an Inducible RGS5 Cell Line

The Tet-Off advanced inducible gene expression system (Clontech) was used to create the RGS5-inducible HeyA8-MDR cell line. The pTRE-Tight dual RGS5-expressing DNA plasmid was constructed by standard restriction enzyme cloning to insert an HA-tagged RGS5 coding sequence cassette (Missouri S&T cDNA Resource Center, Rolla, MO, USA) into the pTRE-Tight Dual vector using the restriction enzymes XbaI and NheI. The constructs were verified by DNA sequencing using specific primers that were designed to recognize the N′-terminus of our Advanced Vector promoter. In order to create the doxycycline inducible cell line, 2.5 × 10^5^ HeyA8-MDR cells were plated in a 6-well dish and transfected at a 2 : 1 ratio (plasmid DNA: Lipofectamine 2000, Life Technologies, Grand Island, NY, USA) with pTet Advanced inducible vector plasmid DNA. Positive clones were selected using G418 (Geneticin, Life Technologies). These stable cells were then cotransfected with pTRE-Tight, Dual HA-RGS5 containing plasmid DNA, and the linear hygromycin marker to enable selection with hygromycin. Positive clones were maintained in paclitaxel, G418, doxycycline, and hygromycin. 

Gene expression was verified in HeyA8-MDR pTet dual HA-tagged RGS5-inducible cells by seeding the cells in a multiple 6-well plates in medium with tetracycline-free fetal bovine serum without doxycycline for 24, 48, and 72 hours. Cells were harvested at the indicated time points, the RNA was isolated and processed for qRT-PCR using primers to detect RGS5 expression (amplicon size: 153 bp) resulting from the Tet-Off Advanced inducible system. The following primers were selected from PrimerBank (http://pga.mgh.harvard.edu/primerbank/), to confirm gene expression using qRT-PCR (RGS5 Fwd: 5′-ATTCAAACGGAGGCTCCTAAAG-3′ and RGS5 Rev: 5′-CACAAAGCGAGGCAGAGAATC-3′). 

### 2.3. BrdU Proliferation Analysis

HeyA8-MDR cells were plated in a 96-well plate at a density of 3,000 cells per well. Half of the plate was grown in medium containing regular fetal bovine serum with doxycycline and the other half doxycycline-free with medium containing tetracycline-free fetal bovine serum. Cells were then incubated at 37°C for 72 hours to allow for RGS5-inducible gene expression. Prior to fixation, HeyA8-MDR cells were pulse-treated for 1 hour with BrdU and then treated for 4 hours with the indicated cell cycle arrest compounds. Cells were then fixed and stained for proliferation and nuclear morphology according to standard procedures from the manufacturer's protocol (BrdU Assay Kit, Millipore). Representative images were taken using the Cellomics ArrayScan VTI HCS Reader (Thermo Fisher Scientific, Waltham, MA, USA) as previously described [24] and are shown here. High-content scanning analysis software was used to determine the average number of cells per field. The data was retrieved from the manufacturer's software and results were plotted with GraphPad Prism (La Jolla, CA, USA).

### 2.4. Animal Model of Ovarian Cancer with Gene Modulation

Six-week-old female athymic nude mice acclimated to the animal facility for 1 week prior to the commencement of the study. Animals were injected intraperitoneally with Extracel containing approximately 5 million cells of either HeyA8-MDR pTet Advanced Vector (*N* = 10) or HeyA8-MDR pTet Dual RGS5 expressing cells (*N* = 10) per 0.2 *μ*L injection. Injected mice were monitored for tumor formation, weight, and stomach circumference. After one week, 100% of mice displayed tumor formation. The animals were monitored over a course of 2 months and euthanized according to the animal use protocol approved by the University of Georgia IACUC committee. The tumor volume (mm^3^) was calculated using the equation tumor volume = (width)^2^ ×  length/2, and then graphed using Prism. The time of survival for each group and overall significance was plotted on a Kaplan-Meier survival curve also using GraphPad Prism. 

### 2.5. Measurement of Vascular Endothelial Growth Factor

At necropsy, blood from all animals was collected in BD microtainer tubes with serum separator (Becton Dickinson Co., Franklin Lakes, NJ, USA). The serum-containing fraction was isolated using centrifugation, placed into glass vials, and frozen immediately. After thawing on ice, the mouse serum was measured for the presence and concentration of vascular endothelial growth factor a mouse VEGF ELISA kit following the manufacturer's protocol (RayBiotech, Inc., Norcross, GA, USA).

### 2.6. Tissue Collection, Histology, and Immunofluorescence

Mice were euthanized according to standard protocols. Visible tumors were dissected from the abdomen, measured for size, and flash-frozen with cryomatrix (Thermo Fisher Scientific) in 2-methylbutane (Sigma) cooled to −140°C. Cryopreserved tumors were cut in 10 *μ*m sections using a Thermo Fisher Scientific cryostat and mounted on microscope slides. Hematoxylin and eosin staining was performed according to standard protocols and imaged using a Leica microscope for pathological evaluation.

For immunofluorescence, tissues were blocked for 30 minutes in 5% normal donkey serum (Jackson ImmunoResearch Laboratories, Inc.,West Grove, PA, USA) in phosphate-buffered saline (PBS) for 30 minutes at room temperature. The tumor sections were incubated in the primary antibody CD31 (1 : 500, BD Pharmingen, San Diego, CA, USA) at 4°C overnight in a humidity chamber, washed with PBS, and detected with the secondary antibody Rhodamine (TRITC)-conjugated AffiniPure F(ab′) 2-Fragment Goat Anti-Rat IgG (H + L) from Jackson ImmunoResearch Laboratories, Inc. (West Grove, PA, USA). DAPI nuclear stain (final 1 : 10,000) was included in the secondary antibody incubation. After thorough washing with PBS, slides were coverslipped with Permount (ThermoFisher Scientific). Immunofluorescence was imaged using an X71 inverted microscope (Olympus, Center Valley, PA) at 20x magnification. Images were resized and adjusted identically using Photoshop (Adobe, San Jose, CA, USA). Overlapping pictures were aligned in Microsoft PowerPoint to generate an image of an entire tumor cryosection. Each compiled tumor section was imported into Image-Pro Express (Media Cybernetics, Bethesda, MD, USA) for analysis. Tumor vascularization was determined by counting the number of complete and partial vessels visible in the tumor section (stained by antibody CD31). Tumor area was calculated using the polygon tool in Image-Pro Express and normalized vascularization was plotted (CD31 vessel counts/tumor area in *μ*m^2^ × 1 × 106).

## 3. Results

### 3.1. Expression of RGS5 Reduces *In Vitro *Proliferation of HeyA8-MDR Ovarian Cancer Cells

**Figure 1:**
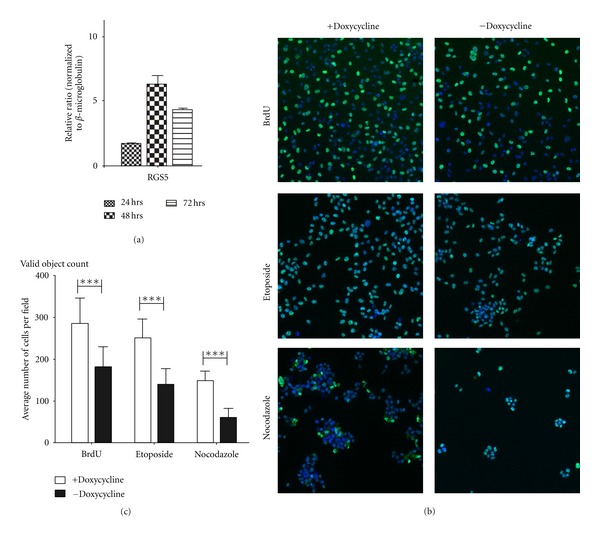
RGS5 inducible expression in HeyA8-MDR cells reduces proliferation. (a) HeyA8-MDR pTet dual RGS5 inducible cells were seeded in a multiple 6-well and cultured in medium with tetracycline-free fetal bovine serum without doxycycline for 24, 48, and 72 hours. Cells were harvested; the RNA was isolated and processed for qRT-PCR using primers to detect RGS5 expression resulting from the Tet-Off Advanced inducible system. Without the presence of doxycycline or other members of the tetracycline antibiotics, RGS5 expression was observed. (b) HeyA8-MDR cells were plated in a 96-well plate at a density of 3,000 cells per well. Half of the plate was grown in medium containing regular FBS with doxycycline and the other half containing doxycycline-free media. Cells were then incubated at 37°C for 72 hours to allow for RGS5-inducible gene expression. Prior to fixation, HeyA8 MDR cells were pulse-treated for 1 hour with BrdU and then treated for 4 hours with the indicated cell cycle arrest compounds. Cells were then fixed and stained for proliferation and nuclear morphology. Representative images taken using the Cellomics ArrayScan VTI HCS Reader and are shown here. (c) High-content scanning analysis software was used to determine the average number of cells per field.

Although RGS5 is a biomarker for tumor vasculature, we sought to understand whether RGS5 itself plays a pathogenic or protective role in ovarian tumor biology. To address this question, we constructed RGS5 in an inducible gene expression system to induce high levels of RGS5 protein when cells were cultured in the absence of the antibiotic doxycycline. The Tet-Off inducible system was necessary because RGS proteins regulate G-protein-coupled receptor signaling cascades, which are required for cancer cells survival and are often critical to cells with oncogenic addictions to survival pathways. When cells were grown in the absence of doxycycline and medium containing FBS free of tetracycline, the expression of RGS5 protein reached ~7 fold after 48 hours and ~4.5 fold after 72 hours (Figure 1(a)).

When we compared the vector control cells (+doxycycline) versus RGS5-expressing HeyA8-MDR cells (−doxycycline), we observed a significant reduction in the number of proliferating cells among the latter group with induced expression of RGS5 (Figure 1(b)). Representative images are shown from high-throughput scanning (see Section 2.3). We next analyzed cancer cell proliferation using automated quantification of cells detected per field after a pulse with bromodeoxyuridine (BrdU), a nucleoside analogue and marker for proliferating cells. Cells engineered to inducibly express RGS5 showed a significant reduction in the number of BrdU-positive proliferating cells (***P < 0.001, comparing +dox with –dox). Treating the cells with either antineoplastic reagents etoposide, a topoisomerase II inhibitor, or nocodazole, an inhibitor of microtubule polymerization, further reduced the average number of cells measured per field (Figures 1(b) and 1(c); ***P < 0.001), although the ratio was similar between the nontreated and treated conditions. As a control, we measured no net change in the mean difference of “target” BrdU fluorescence intensity among the specific cell cycle compounds, suggesting no net bias effect of the fluorescence. Taken together, these data suggest that RGS5 expression reduces the proliferative capacity of HeyA8-MDR ovarian cancer cells.

### 3.2. Expression of RGS5 in an Ovarian Tumor Model Increases Survival

**Figure 2:**
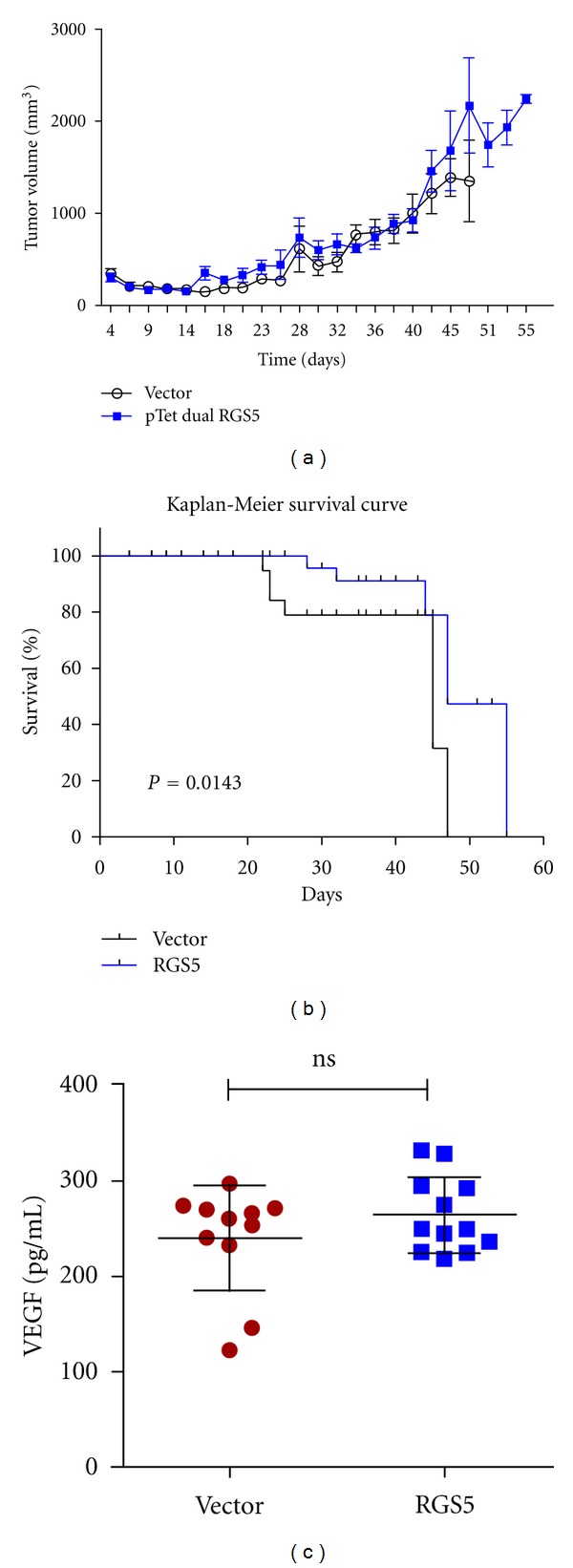
Expression of RGS5 in ovarian tumors did not significantly reduce the rate of growth, but did increase the overall survival time of mice bearing such tumors. (a) Female athymic nude mice were given intraperitoneal injections of Extracel containing approximately 5 million cells of either HeyA8 MDR pTet Advanced Vector (*N* = 10) or HeyA8-MDR pTet Dual RGS5 expressing cells (*N* = 10) per 0.2 *μ*L injection. All mice were monitored for tumor formation and measurements of weight and stomach circumference were routinely taken. The graph plots tumor volume (mm^3^). (b) Mice bearing HeyA8-MDR pTet Dual RGS5-expressing tumors had a significant increase in survival time (in days, *P = 0.0143, compared to their pTet Advanced Vector alone counterparts). (c) Blood was collected from mice at necropsy. The serum-containing fraction was isolated and measured for vascular endothelial growth factor. Results are nonsignificant (ns) between the groups.

Previous studies have examined the absence of RGS5 expression *in vivo* using knockout mice [22]. In contrast with these studies, we created an *in vivo* model of tumorigenesis with inducible expression of RGS5 to measure whether there was an effect on tumor regression. Athymic nude female mice were injected intraperitoneally with tumors containing either the vector alone or RGS5-expressing tumors. We routinely measured tumor volume, but were unable to detect any differences between these groups (Figure 2(a)). In contrast, control animals with empty vector tumors displayed lower body conditioning scores than mice bearing RGS5 tumors and therefore had to be monitored more frequently. Mice bearing RGS5 tumors displayed more active behavior and appeared healthier over a longer period of time with higher body conditioning scores in comparison (data not shown). 

We also measured the difference in survival times between the two groups. The control mice with empty vector tumors began to die (or were euthanized because they reached humane endpoints) at 22 days and all succumbed to disease by 47 days (Figure 2(b)). In comparison, the first mouse from the group bearing RGS5 tumors died in 28 days, and the last two in the group died after 55 days. The results suggest a significant increase in survival time (*P* = 0.0143) with RGS5 expression, although there were no animals that ultimately survived the disease.

Since RGS5 has been shown to be a hypoxia-inducible gene regulated by HIF-1*α* [17], we measured vascular endothelial growth factor (VEGF) in serum. Solid tumors will develop hypoxic regions, which would then upregulate RGS5 and possibly interfere with our results. We chose VEGF because HIF-1 regulates VEGF expression as well as RGS5. If we detected significantly altered levels of VEGF between the groups, then this could indicate a problem with the data. However, there was no significant difference between the serum levels of VEGF between the groups (Figure 2(c)), suggesting that endogenous RGS5 regulation was unchanged. 

### 3.3. Histological Studies Suggest Large Areas of Necrosis in RGS5-Expressing Tumors

**Figure 3:**
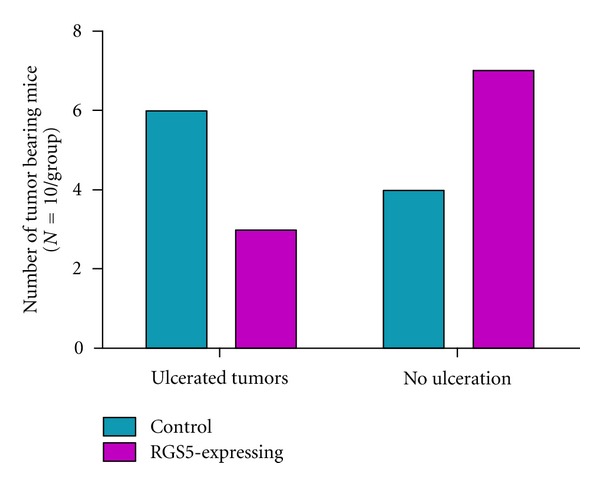
Mice bearing RGS5-expressing ovarian tumors displayed less frequent tumor ulceration. The number of mice with ulcerated tumors observed at necropsy was recorded (*N* = 10 per group). Tumor sizes were relatively similar between the two groups.

We randomly selected tumors from each group for sectioning and histological analysis. Interestingly, the hematoxylin and eosin stained tumor sections showed several important differences between control and RGS5-expressing tumors. In the control group of mice with tumors containing the empty vector, diffuse sheets of malignant cells comprised 70–95% of the sampled tissue, demonstrating extensive involvement of the tumor (Table 1). In contrast, mice bearing tumors expressing RGS5 had regions of necrosis that varied from scattered necrotic areas to broad and large areas of central necrosis. RGS5-expressing tissue was also composed of ~60–90% tumor. In the necrotic areas of RGS5-expressing tumors, pyknotic nuclei and dark eosinophil cytoplasm were observed in the malignant cells along the peripheral areas of the necrotic regions. Finally, the histological tumor samples of the control mice showed areas of skin ulceration, whereas the RGS5-expressing sections did not. This is in agreement with the overall observations where we observed a reduced presence of tumor ulceration in mice bearing RGS5-expressing tumors (Figure 3).

### 3.4. Tumor Angiogenesis

**Figure 4:**
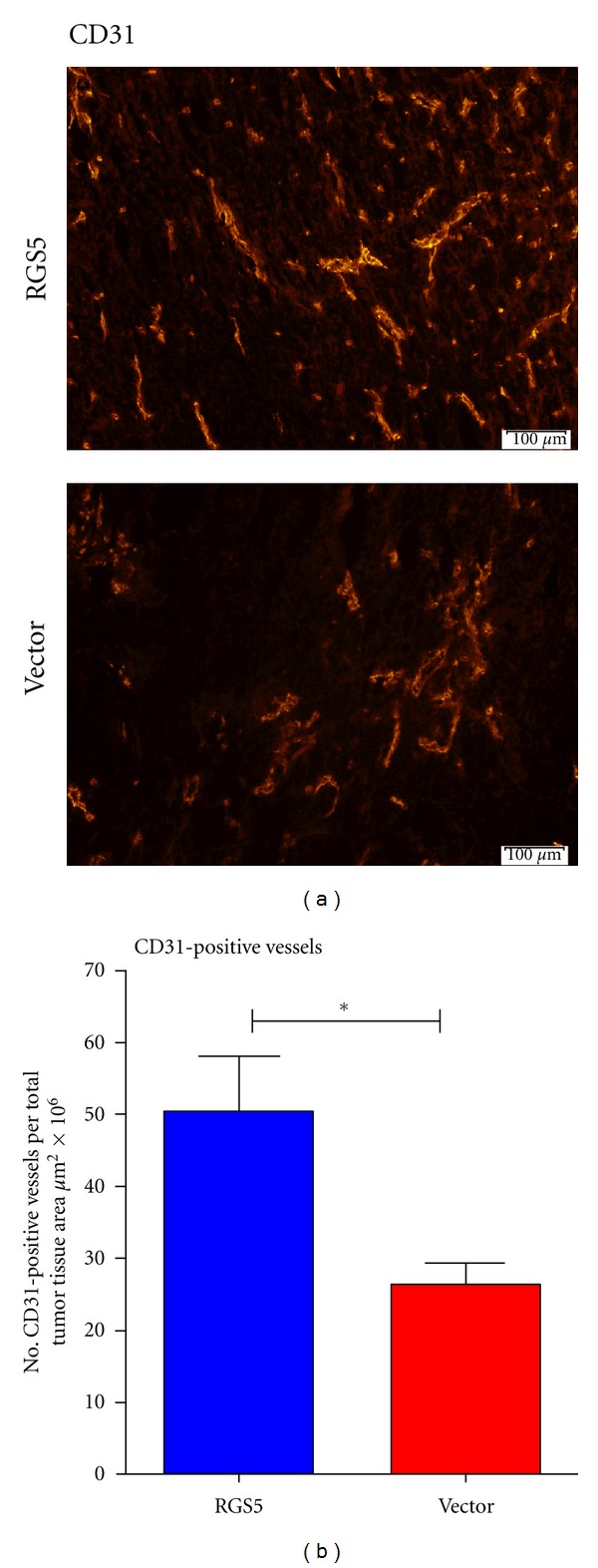
RGS5-expressing ovarian tumors increase the number of CD31-positive vessel-like structures. Tumor specimens were sectioned and prepared on slides for immunofluorescence. (a) Tumor sections were imaged for CD31 expression (red, 20x magnification). (b) CD31-positive vasculature was quantified from compiled whole tumor images (*N* = 4 vector, *N* = 4 RGS5) using CellSens and GraphPad Prism software. **P < 0.01, comparing vector versus RGS5.

To clarify the functional contribution of RGS5 expression in tumor angiogenesis, we randomly selected tumors from each group for sectioning and analysis of positively stained structures of the cluster of differentiation 31 (CD31) or platelet/endothelial cell adhesion molecule 1 (PECAM-1), which is a glycoprotein biomarker expressed on vascular endothelial cells used to assess the degree of angiogenesis. We observed CD31-positive structures in tumor specimens from both groups of animals (Figure 4(a)). The RGS5-expressing tumors displayed a greater frequency of CD31-positive vessel-like structures, compared with the vector-expressing tumors (Figure 4(b), **P < 0.01). This is consistent with the role of RGS5 as a pericyte biomarker that is temporally upregulated during the switch to angiogenesis in malignancy. The data is suggestive that introducing RGS5 into the solid tumor likely influenced the balance of this switch in favor of angiogenesis.

## 4. Discussion 

In this study, we used Tet-off inducible expression to study the role of RGS5, *in vitro* and *in vivo*, in tumor proliferation and pathology. We found that mice bearing RGS5-expressing tumors survived longer than controls and displayed large regions of necrosis within their tumors. They were also less likely to have ulcerated tumors in comparison to control mice. Our study is consistent with previous work that produced RGS5-deficient mice and suggested that RGS5 loss accelerated tumor development, enhanced tumor growth (in the later tumor stages), reduced survival, decreased hypoxia, and decreased vessel permeability [20–22]. 

Hamzah et al. created an RGS5 knockout mouse model using a mix of normal 129 and C57BL/6 mice crossed with the C3H background, which then allowed the assessment of immune function. Although not statistically significant, the RGS5 knockout mouse model resulted in the opening of solid tumors to spontaneous immune effector T-cell infiltration into the tumor parenchyma. In addition, this model showed prolonged survival among tumor-bearing mice with the transfer of activated and specific T cells [20]. Our study used athymic immunodeficient nude mice, which manifest an inhibition of the immune system and thus will not mount an immune response to xenograft injection. It is interesting that we observed vast regions of necrosis in the solid tumors without the immune system modulating this response in RGS5-expressing tumors. This necrosis is likely due to two factors: hypoxia resulting from aberrant tumor vasculature and RGS5 induction of apoptosis [17]. 

Although we observed an increase in the survival time in mice bearing RGS5-expressing tumors, the overall increase in time was modest. Too many chemotherapies and biological agents also achieve mediocre increases in overall survival, leading the industry to focus instead on quality-of-life parameters for measuring drug “successes.” The results of our study dampen enthusiasm for pursuing RGS5 as a single target for therapeutics in tumorigenesis. However, our study does provide support for including RGS5 as one important component of a multicomponent approach to help modulate tumor progression. As the treatment of cancer is a multifaceted discipline, and cytotoxic chemotherapy is combinatorial, momentum for combinatorial biological therapies is also gaining, even among “magic bullet” therapeutics (e.g., imatinib and vemurafenib). The shift is being driven by chemoresistance to therapy, which is relevant in this setting because RGS proteins are involved in chemoresistance [23] as well as hypoxia—a driver of chemoresistance [25]. 

Our study also demonstrates that RGS5 effects active cellular proliferation in HeyA8-MDR cells *in vitro* using the BrdU assay. This result is in contrast with other studies suggesting that the overexpression of RGS5 reduces the rate of growth without affecting cell proliferation [17]. The differences between the two studies are likely the model system. Whereas we are using rapidly proliferating ovarian cancer cells resistant to paclitaxel, the previous study used human umbilical vein endothelial cells. Thus, our model system is highly aggressive, tumorigenic, and drug-resistant with or without RGS5 expression. 

The addition of chemotherapeutic agents (etoposide or nocodazole) to the culture of RGS5-inducible HeyA8-MDR cells further reduced the average number of cells present and proliferating. Although very exciting, the fact that RGS5 has a dual role tumor biology (i.e., vascularization versus tumor growth) makes it unclear how modulation of RGS expression would affect therapy. For example, RGS5 modulation could significantly complicate drug delivery into solid tumor parenchyma. On one hand, RGS5 loss results in normalization of the vasculature *in vivo*, which would allow penetration of T cells and chemotherapy, but otherwise RGS5 loss enhances tumor growth [20]. On the other hand, RGS5 is overexpressed in aberrant tumor vasculature [11], but its expression induces endothelial apoptosis [17], reduces cell proliferation, and increases overall survival. Indeed, in our study the mice bearing RGS5-expressing tumors that showed large areas of central necrosis were not treated with cytotoxic chemotherapy; however, if we had treated these mice with intravenous cisplatin or carboplatin, it is unclear whether these drugs could have reached the tumor parenchyma without a robust vasculature and what the effect on overall survival would have been.

In addition, whether or not angiogenesis is an optimal target for therapy against solid tumors is another growing controversy. Recent studies in glioblastoma hypothesize that vascular normalization improves survival through tumor perfusion [25]. Furthermore, another study of bevacizumab-treated rats bearing human glioblastoma multiforme tumors demonstrated, “a strong and highly significant increase in the number of tumor cells invading the normal brain” [26], suggesting a negative effect on tumor biology in that setting.

In 2011, the Food and Drug Administration revoked its approval of bevacizumab for therapy in metastatic breast cancer because it lacked efficacy and increased the risk for lethal side effects. Some speculated that this review by the FDA was also necessary due to bevacizumab's extraordinarily high yearly cost without the possibility of achieving monotherapeutic cure. For example, bevacizumab is indicated for use as combination therapy in metastatic colorectal cancer, nonsquamous nonsmall cell lung cancer and metastatic renal cell carcinoma and as a single agent for glioblastoma patients based on objective response rate, not survival [27]. Thus, combination regimens with angiogenesis inhibitors have substantially increased the expense of cancer treatment through drug costs and the costs associated with hospitalization for adverse drug responses [28]. A better understanding of the tumor vasculature and its impact on the biology of solid tumors and therapy is needed to address this controversial approach. 

Future studies might explore the roles of combinations of RGS5 with other RGS proteins in ovarian cancer. For example, on chromosome 1q23.3–1q31 there are 5 genes encoding RGS family members (RGS2, RGS4, RGS5, RGS8, and RGS16), and these have overlapping cellular functions [8]. It is unclear whether combinations of these RGS proteins would further modulate the aggressiveness of ovarian cancer cells or tumors in mice. Since RGS proteins turn off signaling cascades from growth factors, future studies could also assess the modulation of these with other proteins affecting G-protein-coupled receptors and receptor inhibitors. Much is left to learn about RGS proteins and their role in tumorigenesis and therapy.

## Figures and Tables

**Table 1:** Histological examination of tumor sections by a pathologist.

Vector controls	Tissue type	Tumor (%)	Necrosis	Mitoses (mm^2^)	Karyorrhexis
1	Mesentery	70	Not identified	<1	Yes
2	Skin	95	Ulcer/focal	<1	Yes
3	Mesentery	>90	Not identified	<1	Yes

RGS5-expressing					

1	Mesentery	>90	Broad area	1	Yes
2	Mesentery	>90	Broad area(s)	1	Yes
3	Mesentery	60	Scattered	1	Yes

## References

[1] Zhou J, Moroi K, Nishiyama M (2001). Characterization of RGS5 in regulation of G protein-coupled receptor signaling. *Life Sciences*.

[2] Chen C, Zheng B, Han J, Lin SC (1997). Characterization of a novel mammalian RGS protein that binds to G*α* proteins and inhibits pheromone signaling in yeast. *The Journal of Biological Chemistry*.

[3] Seki N, Sugano S, Suzuki Y (1998). Isolation, tissue expression, and chromosomal assignment of human RGS5, a novel G-protein signaling regulator gene. *Journal of Human Genetics*.

[4] Xiao B, Zhang Y, Niu WQ, Gao PJ, Zhu DL (2009). Haplotype-based association of regulator of G-protein signaling 5 gene polymorphisms with essential hypertension and metabolic parameters in Chinese. *Clinical Chemistry and Laboratory Medicine*.

[5] Chang YPC, Liu X, Kim JDO (2007). Multiple genes for essential-hypertension susceptibility on chromosome 1q. *American Journal of Human Genetics*.

[6] Faruque MU, Chen G, Doumatey A (2011). Association of ATP1B1, RGS5 and SELE polymorphisms with hypertension and blood pressure in African-Americans. *Journal of Hypertension*.

[7] Cho H, Park C, Hwang IY (2008). Rgs5 targeting leads to chronic low blood pressure and a lean body habitus. *Molecular and Cellular Biology*.

[8] Campbell DB, Lange LA, Skelly T, Lieberman J, Levitt P, Sullivan PF (2008). Association of RGS2 and RGS5 variants with schizophrenia symptom severity. *Schizophrenia Research*.

[9] Smith EN, Bloss CS, Badner JA (2009). Genome-wide association study of bipolar disorder in European American and African American individuals. *Molecular Psychiatry*.

[10] Bondjers C, Kalén M, Hellström M (2003). Transcription profiling of platelet-derived growth factor-B-deficient mouse embryos identifies RGS5 as a novel marker for pericytes and vascular smooth muscle cells. *American Journal of Pathology*.

[11] Berger M, Bergers G, Arnold B, Hammerling GJ, Ganss R (2005). Regulator of G-protein signaling-5 induction in pericytes coincides with active vessel remodeling during neovascularization. *Blood*.

[12] Mitchell TS, Bradley J, Robinson GS, Shima DT, Ng YS (2008). RGS5 expression is a quantitative measure of pericyte coverage of blood vessels. *Angiogenesis*.

[13] Gerlinger M, Rowan AJ, Horswell S (2012). Intratumor heterogeneity and branched evolution revealed by multiregion sequencing. *The New England Journal of Medicine*.

[14] Silini A, Ghilardi C, Figini S (2012). Regulator of G-protein signaling 5 (RGS5) protein: a novel marker of cancer vasculature elicited and sustained by the tumor’s proangiogenic microenvironment. *Cellular and Molecular Life Sciences*.

[15] Papandreou I, Cairns RA, Fontana L, Lim AL, Denko NC (2006). HIF-1 mediates adaptation to hypoxia by actively downregulating mitochondrial oxygen consumption. *Cell Metabolism*.

[16] Warburg O (1956). On respiratory impairment in cancer cells. *Science*.

[17] Jin Y, An X, Ye Z, Cully B, Wu J, Li J (2009). RGS5, a hypoxia-inducible apoptotic stimulator in endothelial cells. *The Journal of Biological Chemistry*.

[18] Weinberg RA (2007). *The Biology of Cancer*.

[19] Weis SM, Cheresh DA (2005). Pathophysiological consequences of VEGF-induced vascular permeability. *Nature*.

[20] Hamzah J, Jugold M, Kiessling F (2008). Vascular normalization in Rgs5-deficient tumours promotes immune destruction. *Nature*.

[21] Manzur M, Hamzah J, Ganss R (2008). Modulation of the “blood-tumor” barrier improves immunotherapy. *Cell Cycle*.

[22] Manzur M, Hamzah J, Ganss R (2009). Modulation of G protein signaling normalizes tumor vessels. *Cancer Research*.

[23] Hooks SB, Callihan P, Altman MK, Hurst JH, Ali MW, Murph MM (2010). Regulators of G-Protein signaling RGS10 and RGS17 regulate chemoresistance in ovarian cancer cells. *Molecular Cancer*.

[24] Jia W, Eneh JO, Ratnaparkhe S, Altman MK, Murph MM (2011). MicroRNA-30c-2* expressed in ovarian cancer cells suppresses growth factor-induced cellular proliferation and downregulates the oncogene BCL9. *Molecular Cancer Research*.

[25] Sorensen G, Emblem K, Polaskova P (2012). Increased survival of glioblastoma patients who respond to antiangiogenic therapy with elevated blood perfusion. *Cancer Research*.

[26] Keunen O, Johansson M, Oudin A (2011). Anti-VEGF treatment reduces blood supply and increases tumor cell invasion in glioblastoma. *Proceedings of the National Academy of Sciences of the United States of America*.

[27] Genentech I, Genentech I (2011). U.S. BL 125085 Supplement: Bevacizumab. *South San Francisco*.

[28] Jackson DB, Sood AK (2011). Personalized cancer medicine—advances and socio-economic challenges. *Nature Reviews Clinical Oncology*.

